# Towards a circuit mechanism for movement tuning in motor cortex

**DOI:** 10.3389/fncir.2012.00127

**Published:** 2013-01-18

**Authors:** Thomas C. Harrison, Timothy H. Murphy

**Affiliations:** Department of Psychiatry, University of British ColumbiaVancouver, BC, Canada

**Keywords:** motor cortex, microcircuitry, projection neurons, layer 5, tuning

## Abstract

The firing rates of neurons in primate motor cortex have been related to multiple parameters of voluntary movement. This finding has been corroborated by stimulation-based studies that have mapped complex movements in rodent and primate motor cortex. However, it has been difficult to link the movement tuning of a neuron with its role within the cortical microcircuit. In sensory cortex, neuronal tuning is largely established by afferents delivering information from tuned receptors in the periphery. Motor cortex, which lacks the granular input layer, may be better understood by analyzing its efferent projections. As a primary source of cortical output, layer 5 neurons represent an ideal starting point for this line of experimentation. It is in these deep output layers that movements can most effectively be evoked by intracortical microstimulation and recordings can obtain the most useful signals for the control of motor prostheses. Studies focused on layer 5 output neurons have revealed that projection identity is a fundamental property related to the laminar position, receptive field and ion channel complement of these cells. Given the variety of brain areas targeted by layer 5 output neurons, knowledge of a neuron's downstream connectivity may provide insight into its movement tuning. Future experiments that relate motor behavior to the activity of neurons with a known projection identity will yield a more detailed understanding of the function of cortical microcircuits.

## Introduction

One of the great achievements in neuroscience is our detailed understanding of the circuitry and function of the visual system. A well-defined anatomical framework and an established parameter space for visual stimulation have expedited research on the computations performed by the visual cortex. A particularly productive approach has been to develop circuit models of the visual cortex based on its multiple input channels and to associate these microcircuits with the macroscopic functional areas of visual cortex (Sincich and Horton, 2005). Canonical circuits may be conserved across all cortical regions, but it is apparent that motor cortex, largely devoid of the granular input layer that has anchored the study of the visual system, must be considered in a unique manner (Poggio and Bizzi, 2004; Shipp, 2005; Shepherd, 2009). Recent experiments have begun to unravel the microcircuitry of the motor cortex using its deep output layers as a reference point. The next step will be to examine how these local microcircuits vary with macroscopic maps of motor function.

Whereas the visual cortex is known to contain multiple overlaid maps of neurons tuned to retinotopic space, ocular dominance, orientation, etc. (Swindale, 2000), the topography of motor cortex is less completely defined. In addition to the widely accepted somatotopic organization of motor cortex (Penfield and Boldrey, 1937), evidence is accumulating for a mapping of movement categories (Graziano et al., 2002; Haiss and Schwarz, 2005; Ramanathan et al., 2006; Harrison et al., 2012). The firing of individual neurons in motor cortex can be related to many parameters of movement, but this tuning is less established and more controversial than in sensory cortex. In this review, we will review circuit properties of neurons in motor cortex that are likely to confer movement tuning in an effort to link local microcircuitry with macroscopic functional maps and motor behavior.

## Movement tuning in motor cortical neurons

The movement tuning of a neuron in motor cortex refers to the relationship between its firing rate and variables such as the speed, direction, joint angle, or endpoint of a movement, typically of the contralateral forelimb. Directional tuning of neurons in motor cortex was first observed in recordings made from awake primates performing a two-dimensional center-out reaching task (Georgopoulos et al., 1982, 1986). The firing rate of a single neuron is coarsely tuned to the direction of arm movement, but the activity of a population of neurons can be linearly transformed into a vector that predicts the speed and direction of arm movement (Georgopoulos et al., 1988; Moran and Schwartz, 1999). However, similar tuning can be demonstrated in monkeys trained to resist externally applied forces in various directions without moving their forelimbs (Kalaska et al., 1989). Movement tuning can also be altered by the changing the posture of the forelimb (Scott and Kalaska, 1995). For these reasons, there has been debate as to whether preferred movement directions are indeed a fundamental property of motor cortex or an epiphenomenon emerging from activity more closely related to control of the peripheral musculature (Todorov, 2000; Scott, 2004). It has also been argued that dynamic neuronal activity encodes movement trajectories rather than instantaneous variables such as direction, speed, or force (Hatsopoulos et al., 2007; Reimer and Hatsopoulos, 2009; Churchland et al., 2012). Regardless of the theoretical framework, both kinetic and kinematic information from motor cortex has been productively exploited in the development of brain-machine interfaces that control the movement of computer cursors, artificial limbs, or paralyzed muscles (Wessberg et al., 2000; Hochberg et al., 2006; Chestek et al., 2007; Velliste et al., 2008; Ethier et al., 2012).

## Macroscopic mapping of movement in motor cortex

The motor cortices of primates (Leyton and Sherrington, 1917; Penfield and Boldrey, 1937; Rizzolatti and Luppino, 2001; Dum and Strick, 2002; Gharbawie et al., 2011a) and rodents (Neafsey and Sievert, 1982; Li and Waters, 1991; Tennant et al., 2010) have long been recognized to possess a topographic map of body parts. Beyond this broad somatotopic parcellation, finer structure has been proposed to exist in motor cortex. The minimal stimulus parameters adopted by practitioners of intracortical microstimulation (ICMS) mapping led to the interpretation of motor cortex as a mosaic of individual columns, each controlling a single muscle in the periphery (Asanuma, 1975). This hypothesis has since been refuted based on electrophysiological and anatomical evidence of multiple colonies of cortical neurons that are distributed broadly throughout cortex yet innervate a single spinal motoneuron (Jankowska et al., 1975; Rathelot and Strick, 2006). Furthermore, individual primate corticospinal neurons target multiple motoneurons and can facilitate or suppress several muscles simultaneously (Shinoda et al., 1981; Cheney et al., 1985). In rodents, all descending input is received by spinal interneurons and then relayed to motoneurons that innervate muscles, adding an additional layer of processing between cortex and the musculature. Finally, EMG-based mapping in primates and rodents has revealed substantial overlap of muscle representations in motor cortex (Donoghue and Wise, 1982; Donoghue et al., 1992; Park et al., 2001; Ayling et al., 2009).

In contrast to the strictly somatotopic view of motor cortex obtained by mapping with brief, low-intensity stimuli, experiments with prolonged electrical or optogenetic stimulation have reported an organization of motor cortex output based on movement direction or category (Graziano et al., 2002, 2005; Haiss and Schwarz, 2005; Stepniewska et al., 2005; Ramanathan et al., 2006; Harrison et al., 2012). These stimulation-based experiments broaden the definition of movement tuning in motor cortex, which has traditionally been based on recordings made from neurons during reaching behavior (Georgopoulos et al., 1982). Moreover, they corroborate the clustering of preferred movement directions in motor cortex reported from electrophysiological recordings in primates (Amirikian and Georgopoulos, 2003; Ben-Shaul et al., 2003; Georgopoulos et al., 2007). Directional tuning has also been detected in human motor cortex using functional magnetic resonance imaging (Eisenberg et al., 2010). Given that movement tuning was observed despite the relatively coarse spatial resolution of functional magnetic resonance imaging, clusters of similarly tuned neurons are likely to exist in human motor cortex.

The complex topography of motor cortex could reflect the reduction of multiple dimensions of information onto the two-dimensional cortical surface (Aflalo and Graziano, 2006), reminiscent of the multiple feature maps overlaid onto primary visual cortex (Swindale et al., 2000). Although motor maps may contain clusters of similarly tuned neurons, the level of detail currently detected in these maps is coarser than in sensory maps (Bonhoeffer and Grinvald, 1993; Schreiner and Winer, 2007). For example, calcium imaging has a revealed clustering of neuronal tuning properties in layer 2/3 neurons of rodent motor cortex (Dombeck et al., 2009), but this is less pronounced than that of orientation maps in cat visual cortex (Ohki et al., 2005). Whether this difference is attributable to differences between the species, the nature of the mapped parameter or the cortical region is an open question.

## Origins of movement tuning

Movement tuning is defined by firing rate and must ultimately arise from the pattern of input that drives a particular neuron to fire. As in other cortical areas, local inhibitory neurons in motor cortex are hypothesized to act in concert with excitatory neurons to shape the tuning of downstream cells (Shapley et al., 2003; Georgopoulos and Stefanis, 2007; Merchant et al., 2008, 2012). Electrophysiological recordings have found that fast-spiking interneurons in motor cortex exhibit tuning profiles that are broader than pyramidal neurons, suggesting that they may contribute to movement tuning by restricting all but the most excited neurons from firing (Isomura et al., 2009). In both rodents and primates, inhibitory neurons increase their firing rates throughout movement preparation and execution, suggesting that they are likely to be involved in shaping movments rather than gating them through a sudden release of inhibition (Isomura et al., 2009; Kaufman et al., 2010). Finally, dendritic gating and amplification may provide an additional means for the establishment of tuning beyond passive summation of inputs (London and Hausser, 2005; Harnett et al., 2012; Lee et al., 2012; Xu et al., 2012). Identifying and characterizing the many inputs that impart tuning to motor cortex output neurons seems as daunting now as when Ramón y Cajal lamented the “impenetrable thickets” of cortical networks (Ramón y Cajal, 1989). Fortunately, since excitatory connectivity within cortical microcircuits is specified by the identities of both the pre- and post-synaptic cells, knowledge of the projection identity of motor cortical neurons provides an indication of the source of their inputs (Brown and Hestrin, 2009; Anderson et al., 2010). This makes the output layers of motor cortex a useful starting point for circuit analysis akin to the input layers of visual cortex (Shepherd, 2009).

## Relating movement tuning to microcircuit properties

The fact that recordings from motor cortex can extract useful kinematic information for the control of neural prostheses suggests that the activity of neurons in the motor cortex encodes information relevant to the direction of intended movement (Chapin et al., 1999; Chadwick et al., 2011; Collinger et al., 2012). It remains to be determined how the circuit properties of cortical neurons confer their movement tuning. The majority of our knowledge about the firing properties of motor cortical neurons and movement tuning has come from primate studies, where relating neuronal activity to such microcircuit variables can be difficult (Sheets and Shepherd, 2011). The microcircuitry of motor cortex is now being studied intensively in rodents (Isomura et al., 2009; Anderson et al., 2010; Matyas et al., 2010; Mao et al., 2011), which possess many experimental advantages but also differ from the primate motor system in terms of thalamic (Aldes, 1988) and intracortical connectivity (Keller, 1993), the relative thickness of cortical layers (Hutsler et al., 2005), and dopaminergic input (Berger et al., 1991). Rodents also lack corticomotoneurons, a class of corticospinal neuron that synapse directly onto motoneurons rather than engaging local spinal circuitry (Lemon, 2008). An attempt to link motor microcircuits with movement tuning must draw upon the relative advantages of multiple animal models, while acknowledging the differences between them. In both primates and rodents, neurons can be classified by a set of inter-related attributes, including the region of cortex that they inhabit laminar position, morphology, and their complement of transmitters and receptors.

The relationship between a neuron's movement tuning and its location within cortex is not well-defined. The existence of multiple movement maps and the broad distribution of motor-related neurons, particularly in primate motor cortex (Gharbawie et al., 2011b), makes it difficult to predict based on cortical position whether a neuron is likely to possess movement tuning. Recordings from M1 provide the most useful signal for neural prostheses (Carmena et al., 2003; Vargas-Irwin et al., 2010), but a greater proportion of neurons in PMv possess “extrinsic-like” tuning to arm movement independent of posture (Kakei et al., 2001). Better established is the link between movement tuning and cortical depth. The concept of the cortical column has been applied to movement tuning in motor cortex, with consistent tuning reported across radial depths of ~500 mm in primates (Ben-Shaul et al., 2003; Georgopoulos et al., 2007). Although signals useful for the control of brain machine interfaces can be extracted without penetrating the cortex (Wolpaw and McFarland, 2004), they are strongest in layers 5–6 (Parikh et al., 2009). Microstimulation studies have found movements to be most easily evoked from these deep cortical layers (Donoghue and Wise, 1982; Neafsey and Sievert, 1982; Young et al., 2011). Selective stimulation of ChR2-expressing neurons located predominantly in layer 5B yields a motor map subdivided by movement direction that persists after pharmacological blockade of intracortical glutamate receptors (Harrison et al., 2012). Taken together, these observations suggest that movement-tuned neurons in motor cortex are present in the deep cortical layers.

In motor cortex, as in all cortical areas, the layer occupied by a neuron's soma is closely related to its projection identity (Hooks et al., 2011; Mao et al., 2011). Neurons in the superficial cortical laminae (2/3) form connections within their layer and send strong projections to layer 5 (Weiler et al., 2008). Layer 2/3 neurons, theorized to selectively amplify inputs to a cortical region (Douglas and Martin, 2004; Weiler et al., 2008; Adesnik and Scanziani, 2010), also possess movement tuning (Merchant et al., 2008; Dombeck et al., 2009). Connectivity in this pathway is determined by both the projection identity and radial position of the recipient neuron within layer 5 (Anderson et al., 2010). The projection identity of layer 5 neurons is also linked with their intralaminar connectivity (Brown and Hestrin, 2009; Kiritani et al., 2012), morphology (Gao and Zheng, 2004) and intrinsic electrophysiological properties (Hattox and Nelson, 2007; Sheets et al., 2011). Therefore, the projection identity of a motor cortical neuron can be related to microcircuit properties including its receptive field and its excitability, which together determine a neuron's tuning properties (Lee et al., 2012).

## Projection identity and movement tuning

Just as the response properties of granular neurons in visual cortex are largely derived from their inputs (Ferster and Miller, 2000; Huberman et al., 2008), it follows that the tuning of corticofugal neurons in motor cortex might be related to their outputs. Layer 5 pyramidal neurons are likely to be mediators of movement tuning within a columnar microcircuit since they constitute the majority of the cortical output pathway. Layer 5 neurons are a heterogeneous population and project to many regions, including cortex, thalamus, brainstem, basal ganglia, and spinal cord (Veinante and Deschênes, 2003; Kiritani et al., 2012). Layer 5 can be further subdivided into layers 5A and 5B, each with characteristic gene expression, receptive field, and projections (Lund et al., 1988; Manns et al., 2004; Anderson et al., 2010; Mao et al., 2011). Layer 5B contains a preponderance of corticospinal neurons (Anderson et al., 2010) that innervate the muscolotopically organized spinal cord (Levine et al., 2012), which in turn orchestrates the synergistic activation of the musculature to achieve movements in stereotyped directions (Bizzi et al., 2002). In the primate corticospinal tract, there is also a group of corticomotoneuronal cells that contact spinal motoneurons directly and whose cell bodies in layer 5 are clustered within a region of motor cortex that is hypothesized to have evolved relatively recently to control fine movements of the distal musculature (Rathelot and Strick, 2009). Not only are these cells likely to possess movement tuning, it is possible that details of their tuning could be predicted based on the motoneuron that they innervate.

Corticostriatal neurons are primarily found in layer 5A. As with corticospinal neurons, sub-types of corticostriatal neurons may have distinct movement tuning depending on their specific projection identity. Pyramidal (PT) type corticostriatal axons synapse in the ipsilateral striatum before continuing on toward the spinal cord. This differentiates them from intratelencephalic (IT) type corticostriatal neurons which cross the midline to project to the striatum and contralateral cortex but do not leave the telencephalon. IT and PT type neurons preferentially innervate direct and indirect pathway neurons of the striatum, respectively (Reiner et al., 2010). Corticostriatal connections are altered by the process of learning to control a neuroprosthetic device, evidenced by an NMDA receptor dependent increase in coherence beteween motor cortex and the striatum (Koralek et al., 2012).

Neurons forming the corticospinal tract are predisposed to possessing movement tuning, but other descending pathways such as the rubrospinal tract are also heavily involved in motor control (Lemon, 2008). Nor is the corticospinal tract exclusively motor-related, with only 55% of primate corticospinal neurons directly facilitating muscle activity (Lemon et al., 1986). Spinal motoneurons receive synaptic input from many sources, meaning that even direct corticomotoneurons have a variable influence on the muscles they innervate (Yanai et al., 2007). Rodents lack corticomotoneurons, making the contribution of the network of spinal interneurons to movement tuning a further consideration (Levine et al., 2012). The complexity of descending motor pathways provides an explanation for the observation that activity in motor cortex can become uncoupled from movement during sleep, motor imagery, or control of brain machine interfaces (Schieber, 2011). In fact, the process of learning to control a brain–machine interface causes widespread changes in the preferred direction tuning of neurons throughout the motor cortex (Ganguly et al., 2011), with the greatest increase in performance coming from neurons in the supplementary motor area (Carmena et al., 2003).

## Conclusions and future directions

**Figure 1:**
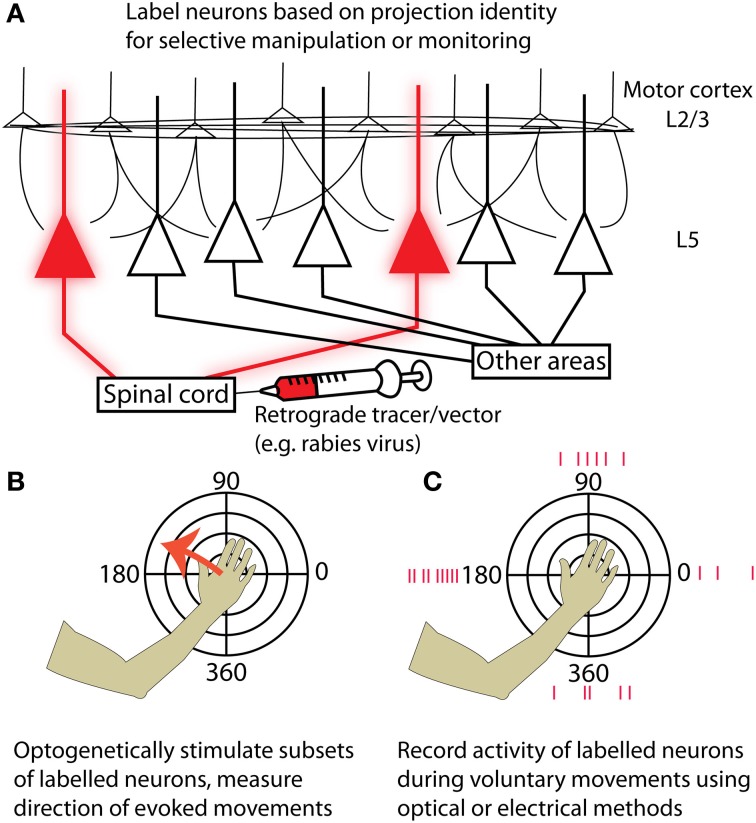
**Strategies for targeting neurons in motor cortex based on their projection identity. (A)** Injecting a retrograde tracer or viral vector into a region downstream of motor cortex (for example, the spinal cord) will label neurons in motor cortex that project to that area. **(B)** By expressing optogenetic activators such as Channelrhodopsin-2, neurons of the targeted projection class can be selectively activated while resulting movements are measured. Motor maps generated by stimulating specific projection classes can be tested for topographies of evoked movement direction and compared with maps from other projection classes. **(C)** Alternatively, the activity of labeled neurons can be measured while an experimental animal makes voluntary movements in different directions. This could be accomplished either with electrical recordings or by imaging genetically encoded calcium or voltage indicators. Again, preferred movement directions can be examined at different cortical locations for neurons in a given projection class, and different projection classes can be compared by performing retrograde injections in multiple structures targeted by cortical output neurons.

Movement tuning is a well-documented yet controversial property of motor cortex that has been successfully exploited for direct cortical control of neural prostheses (Hochberg et al., 2006; Velliste et al., 2008). The deep layers of motor cortex contain the most useful signal for these brain machine interfaces (Parikh et al., 2009) and are also the site from which movements can most easily be evoked by stimulation (Young et al., 2011). Neurons in layer 5 form outputs to a variety of structures involved in motor control, including the striatum, brain stem, and spinal cord. The advent of new experimental tools for combined anatomical and physiological circuit tracing based on retrograde transmission of Cre-fused wheat germ agglutinin (Gradinaru et al., 2010) or modified rabies virus (Wickersham et al., 2007; Wall et al., 2010; Apicella et al., 2012; Kiritani et al., 2012) has made it possible to label neurons based on projection identity. Although some of these tools are currently used primarily in mice, efforts are underway to apply them in primate models (Diester et al., 2011). This will make it possible to both monitor and manipulate the activity of specific motor output pathways (e.g., corticospinal) to isolate their contribution to motor behavior (Figure 1). It will then become possible to identify the upstream circuit mechanisms that enable flexible control of these pathways during motor imagery or behavior. These experiments will dramatically enhance our understanding of the cortical motor system and the nervous system as a whole.

### Conflict of interest statement

The authors declare that the research was conducted in the absence of any commercial or financial relationships that could be construed as a potential conflict of interest.
